# Dynamics of Microbial Communities in Phototrophic Polyhydroxyalkanoate Accumulating Cultures

**DOI:** 10.3390/microorganisms10020351

**Published:** 2022-02-03

**Authors:** Juliana R. Almeida, Joana C. Fradinho, Gilda Carvalho, Adrian Oehmen, Maria A. M. Reis

**Affiliations:** 1 Associate Laboratory i4HB—Institute for Health and Bioeconomy, NOVA School of Science and Technology, NOVA University Lisbon, 2829-516 Caparica, Portugal; jro.almeida@campus.fct.unl.pt (J.R.A.); amr@fct.unl.pt (M.A.M.R.); 2 UCIBIO—Applied Molecular Biosciences Unit, Department of Chemistry, NOVA School of Science and Technology, NOVA University Lisbon, 2829-516 Caparica, Portugal; g.carvalho@uq.edu.au (G.C.); a.oehmen@uq.edu.au (A.O.)

**Keywords:** phototrophic mixed cultures (PMC), phototrophic purple bacteria (PPB), polyhydroxyalkanoates (PHA), feast and famine (FF), permanent feast (PF), domestic wastewater, DNA sequencing, fluorescence in situ hybridization (FISH), microbial communities

## Abstract

Phototrophic mixed cultures (PMC) are versatile systems which can be applied for waste streams, valorisation and production of added-value compounds, such as polyhydroxyalkanoates (PHA). This work evaluates the influence of different operational conditions on the bacterial communities reported in PMC systems with PHA production capabilities. Eleven PMCs, fed either with acetate or fermented wastewater, and selected under either feast and famine (FF) or permanent feast (PF) regimes, were evaluated. Overall, results identified Chromatiaceae members as the main phototrophic PHA producers, along with *Rhodopseudomonas*, *Rhodobacter* and *Rhizobium*. The findings show that Chromatiaceae were favoured under operating conditions with high carbon concentrations, and particularly under the PF regime. In FF systems fed with fermented wastewater, the results indicate that increasing the organic loading rate enriches for *Rhodopseudomonas*, *Rhizobium* and Hyphomicrobiaceae, which together with *Rhodobacter* and Chromatiaceae, were likely responsible for PHA storage. In addition, high-sugar feedstock impairs PHA production under PF conditions (fermentative bacteria dominance), which does not occur under FF. This characterization of the communities responsible for PHA accumulation helps to define improved operational strategies for PHA production with PMC.

## 1. Introduction

The current over-exploitation of natural resources, along with the release of polluting waste streams, are causing an unprecedented impact on the climate and ecosystems [1]. Coupling environmental protection with economic growth requires the implementation of sustainable practices, which include the valorisation of wastes and their reintegration into the process chain, under the so-called circular economy. Bioprocesses can contribute to this valorisation, and agricultural wastes, flue gases, domestic and industrial wastewaters are examples of waste streams that have been targeted for valorisation through e.g., resource recovery, bioconversion to new value-added compounds and energy production [2,3]. By exploring the aptitudes of different microorganisms, a variety of biological systems can be applied to each waste stream scenario. In particular, phototrophic purple bacteria (PPB) have been studied and employed in biological processes that aim at the valorisation of waste streams [4].

PPB are some of the most versatile bacteria, with a very flexible metabolism, which allows them to grow under phototrophic conditions in the light and under chemotrophic conditions in the dark, either heterotrophically or autotrophically (fixating CO_2_), using oxygen or inorganic molecules as terminal electron acceptors in respiratory processes [5]. Their capacity to thrive under photoheterotrophic conditions makes them suitable for applications in organic waste-stream valorisation, including resource recovery from wastewaters [6] and the production of valuable compounds such as single-cell protein, pigments, coenzyme Q-10 and polyhydroxyalkanoates (PHA) [7,8].

PHAs are biopolymers produced and internally accumulated by several microorganisms as carbon and energy reserves. Furthermore, PHAs present thermoplastic properties whilst being completely biodegradable if disposed in nature. For this reason, they have been proposed as a sustainable alternative to the conventional non-degradable petrochemical plastics [9]. Currently, commercialized PHA is typically produced from aerobic single-strain systems fed with defined media and operated under aseptic conditions [10]. These operating requirements not only contribute to high polymer production costs, but also limit the utilization and valorisation of complex organic streams that are wasted by our society on a daily basis. In contrast, mixed microbial cultures (MMC) are robust under fluctuating feedstock compositions, being capable of converting cheap, low-grade, complex wastes into PHA, under non-sterile environments [11]. These simple operating requirements can contribute to decreased polymer production costs while enabling the valorisation of a multitude of organic wastes. Furthermore, MMCs enable the production of polymers with different compositions and properties, and thus with a wide range of applications [11].

PHA production by PPB has been extensively reported for single-strain systems reviewed in [12,13] and to a lesser extent, for phototrophic mixed cultures (PMC), either using synthetic or real feedstocks, such as fermented cheese whey, food waste, domestic and winery wastewater reviewed in [13,14]. In order to attain high PHA accumulations in PMC, it is essential to enrich the culture in PHA-accumulating bacteria. This can be achieved by operating the systems under different selective pressures, such as transient availability of carbon (feast and famine regime—FF), or reductive stress that occurs under permanent organic carbon availability (permanent feast regime—PF). In the first case, the FF regime selects for bacteria that store PHA as carbon reserves during short feast phases for utilisation during long famine phases [15]. In the second case, the PF regime selects for bacteria that store PHA as an electron sink to balance the cell internal redox state [16]. Each regime impacts the bacterial community in its unique way, but it also impacts the microalgae content of the culture. Indeed, in the FF regime, the long illuminated famine phase allows the growth of autotrophic microalgae that produce oxygen, which is used by PPB to consume the stored PHA. In contrast, the PF regime has the continuous presence of organic carbon, which favours PPB over microalgae, minimizing oxygen production and leading to a much more anaerobic environment (ORP −300 to −400 mV) in comparison to FF regimes [14].

Although some studies have reported the PHA production capacity of different PMCs operated under diverse feedstock and operating conditions [13], few have analysed the composition of the microbial communities developed in each system. In the few studies that reported any PMC microbial community characterization, the identification of the organisms was limited to very generic groups like Alphaproteobacteria or Gammaproteobacteria classes [15,17]. For the more mature field of PHA production with aerobic MMC systems, some studies have already correlated operational conditions with the microbial community structure and associated PHA production performance [18,19]. Here, detailed characterization is often reported, where several genera and species of aerobic bacteria were identified as major PHA producers, such as *Paracoccus*, *Azoarcus*, *Thauera, Amaricoccus*, *Zoogloea* and *Plasticicumulans acidivorans.* For phototrophic systems, studies on microbial compositions were reported for PPB-enriched systems designed for wastewater treatment and resource recovery. In addition to a non-phototrophic flanking community, purple bacteria of the genera *Rhodopseudomonas*, *Rhodobacter*, *Allochromatium, Blastochloris* and *Rhodoplanes* could be found [20,21,22]. However, these cultures were not subjected to PHA-producing selective pressures. Therefore, to efficiently develop PMC systems for PHA production, it is critical to understand which organisms are most involved in these processes, and thus far, a systematic collection and evaluation/discussion of the microbial communities present in these systems is yet to be performed. This work proposes to deepen current knowledge about the microbial communities within PHA-producing PMCs through DNA sequencing, fluorescence in situ hybridization (FISH), Nile blue staining and morphological observations of several reported PMC systems designed for PHA production. Links between the microbial communities and their PHA production performance are also established in this paper. It is expected that the identification of the microbial groups with higher PHA storage capacity and their thriving conditions will allow the development of phototrophic mixed microbial systems with increased polymer productivity.

## 2. Materials and Methods

### 2.1. Phototrophic Mixed Cultures Operation

This study analyses the bacterial communities of reported phototrophic mixed culture (PMC) systems that were enriched in phototrophic purple bacteria (PPB) for the production of PHA. For simplification, these systems were divided into two sets: one set regarding the operation of PMCs fed with synthetic medium, where acetate was the sole carbon source (Table 1, cultures S1 to S5) [15,16,17,23] and a second set, regarding the operation of PMCs that were fed with a real waste feedstock composed of a fermented mixture of domestic wastewater supplemented with 1% *v*/*v* molasses (Table 1, cultures R1 to R6) [24]. A local pond sediment was the original inoculum of cultures S1 to S5, and R0 was the original inoculum used to seed cultures R1 to R6 and was collected from a high-rate algal pond treating domestic wastewater at El Torno WWTP (Aqualia) at Chiclana, Andalucia, Spain. For both sets, the PMCs were operated in sequencing batch reactors (SBR), artificially illuminated with halogen lamps. The synthetically fed systems S1 to S5 were operated in closed photobioreactors at 30 °C, pH controlled at 6.5, under argon sparging (to prevent surface aeration). The real-waste-fed systems R1 to R6 were operated in open photobioreactors at 25 °C and in a pH range of 6–7. Alongside with these operational settings, the PMCs were subjected to different operating conditions, namely selection strategy (feast and famine—FF or permanent feast—PF), cycle length, light transience and intensity, organic loading rate (OLR) and sludge retention time (SRT). Table 1 summarizes the operating conditions of each culture analysed in this work and their respective performance in terms of biomass and PHA production.

### 2.2. DNA Sequencing and Taxonomy Assignment

Biomass samples were collected from each phototrophic system (one sample per condition) and stored at −20 °C. DNA extraction and sequencing were performed by DNA Sense (Aalborg, Denmark) on a MiSeq (Illumina, San Diego, CA, USA).

DNA extraction was performed using the standard protocol for FastDNA Spin kit for Soil (MP Biomedicals, Santa Ana, CA, USA) with some alterations. To begin, 500 μL of sample, 480 μL sodium phosphate buffer and 120 μL MTbBuffer were added to a Lysing Matrix E tube. Bead beating was performed at 6 m/s for 4 × 40 s [25]. DNA concentration was measured using Qubit dsDNA HS/BR Assay kit (Thermo Fisher Scientific, Waltham, MA, USA).

Sequencing libraries for the bacterial 16S rRNA gene variable region 1–3 (bV13-A) were prepared by a custom protocol according to [26] using the specific primers [27F] AGAGTTTGATCCTGGCTCAG and [534R] ATTACCGCGGCTGCTGG [27]. The resulting sequencing libraries were purified using the standard protocol for CleanNGS SPRI beads (CleanNA, Waddinxveen, The Netherlands), and gel electrophoresis using Tapestation 2200 and D1000/High sensitivity D1000 screentapes (Agilent, Santa Clara, CA, USA) was used to validate the product size and purity of a subset of sequencing libraries. The purified samples were paired-end sequenced (2 × 300 bp) on a MiSeq (Illumina) using a MiSeq Reagent kit v3 (Illumina) according to the standard guidelines.

Forward and reverse reads were trimmed for quality using Trimmomatic v. 0.32 [28] with the settings SLIDINGWINDOW:5:3 and MINLEN: 275 and posteriorly merged using FLASH v. 1.2.7 [29] with the settings -m 10 -M 200. The trimmed reads were dereplicated and formatted for use in the UPARSE workflow [30]. OTU abundances were estimated using the usearch v. 7.0.1090-usearch_global command and taxonomy was assigned using the uclust classifier as implemented in the assign_taxonomy.py script in QIIME [31] and the MiDAS database v. 2.1.3 [32], which is a curated database based on the SILVA database, release 123 [33]. The results were analysed in R v. 4.1.0 [34] via RStudio IDE (1.4.1717) and using the ampvis package v. 2.7.8 [25].

### 2.3. Fluorescence In Situ Hybridization (FISH) and Morphological Observation

Biomass samples (one sample per condition) were examined microscopically for morphological observation, visualization of intracellular PHA granules (through Nile blue staining) and for bacterial community analysis by fluorescence in situ hybridization (FISH). Nile blue staining was performed on wet biomass according to the method in [35]. For FISH analysis, biomass samples were previously fixed with 4% paraformaldehyde (PFA) or ethanol, as described by [36].

The oligonucleotide probes were applied with a fluorochrome Cy-3, along with a FitC-labeled EUBMIX probe for all Bacteria (EUB338 and EUB338-II and III). The specific probes used were: ARC915 for Archaea, ALF969 for Alphaproteobacteria, BET42a for Betaproteobacteria, GAM42a for Gammaproteobacteria, Delta495a for Deltaproteobacteria. For identification of purple bacteria, GRb was used to identify *Rhodobacter* and *Roseobacter*, Rhodo-2 for Rhodospirillaceae family and Rhodopseud was used to identify *Rhodopseudomonas*. For identification of sulphate reducers, DSBAC357, DSB706 and DSV687 were applied for Desulfobacteraceae, Desulfobulbaceae and Desulfovibrionales, respectively. The RHC439 probe was used for the identification of the Rhodocyclaceae family. Concerning known PHA accumulating bacteria, within the Rhodobacteraceae family PAR651 for *Paracoccus* and AMAR839 for *Amaricoccus* were assessed. Within the Rhodocyclaceae family Azo644 for *Azoarcus*, Thau832 for *Thauera* and ZRA23a for *Zoogloea* were applied. The LGC354 probe was applied for Firmicutes detection. The probe set GAOmix (GBG2 + GAOQ989) was used to verify the existence of glycogen-accumulating microorganisms, namely Candidatus *Competibacter phosphatis*. Additionally, the probe set PAOmix (PAO462 + PAO651 + PAO846) was employed for Candidatus *Accumulibacter Phosphatis*. Details on oligonucleotide probes are available at probeBase [37]. A Zeiss Imager D2 epifluorescence microscope was used for the microscopic observations of the biomass samples.

## 3. Results

### 3.1. DNA Sequencing and Taxonomic Groups Identification

The analysis of the gene amplicon sequences targeting the bacterial 16S rRNA gene variable region 1–3, combined with taxonomic classification, allowed us to estimate the abundance of different microbial groups for each PMC community. Table 2 and Table 3 summarize the main taxonomic groups identified for the PMC selected with synthetic acetate feeding and for the PMC selected with a fermented mixture of domestic wastewater supplemented with molasses, respectively (more detailed information on less abundant groups can be found in supplementary material Tables S1 and S2).

Regarding the PMC fed with synthetic media (Table 2, S1 to S5), despite some occasional occurrence of organisms from other diverse phyla (Actinobacteria, Bacteroidetes, Chloroflexi and Cyanobacteria), the sequencing results show that the majority of the microorganisms were members of the phylum Proteobacteria, with an abundance of over 80% for each sample. Within this phylum, the identified populations belonged mostly to the Alphaproteobacteria class, with a greater incidence of the families Bradyrhizobiaceae, Rhizobiaceae and Hyphomicrobiaceae. Specifically, the bacterial genus Blastochloris (Purple non-sulphur bacteria—PNSB [38]) was particularly dominant in cultures S1 to S4, operated with a feast and famine (FF) regime. Additionally, the class Gammaproteobacteria, which was detected in all cultures, was considerably abundant in the permanent feast (PF) culture S5, particularly with members of the Chromatiaceae family (Purple sulphur bacteria—PSB [5].

Regarding the PMCs fed with fermented domestic wastewaters (Table 3, R1 to R6), the enriched microbial communities showed a broader diversity of populations, from various phyla, than the acetate-fed cultures. While Proteobacteria was the key phylum identified in samples R0, R3 and R4 to R6 (over 51% abundance; cf. supplementary material Table S2), Firmicutes and Actinobacteria were dominant in cultures R1 and R2, respectively (cultures that were operated under a PF regime). Firmicutes (11%) and Actinobacteria (20%) were also well-represented in the inoculum R0 culture (Table S2). Within the Proteobacteria phylum, the greatest abundance was registered in the Alphaproteobacteria class, with a family occurrence similar to what was observed in the synthetically fed cultures, but with the additional genera information on the presence of the PNSB *Rhodopseudomonas* in R3 (PF regime), *Rhizobium* in R3 and R6 (PF and FF regime), the PNSB *Rhodobacter* in all cultures and *Paracoccus* in R4 and R5 (FF regime). Interestingly, a high presence (>60%) of Rhodocyclaceae family members (Betaproteobacteria class) was found in R5 (FF regime with sugar presence). Gammaproteobacteria class members were found to be either absent (R3) or with low abundance in the remaining microbial communities.

### 3.2. Fluorescence In Situ Hybridization (FISH)

Parallel to the taxonomic groups’ identification through DNA sequencing, FISH was also performed to help to identify the microbial populations in each culture. Table 4 aggregates the FISH results reported for cultures S2 to S5 [15,16,17,23] and includes results from this work for S1 and R1 to R6. (See Table S3 for results of the GAOmix and PAOmix probes and Figure S1 for graphical visualization of FISH results of samples R1 to R6). 

Considering the probes employed in these studies, FISH results indicate that the bacterial domain of all synthetically fed PMCs was mostly represented by Alphaproteobacteria and Gammaproteobacteria, with a slight dominance of the latter. The presence of PNSB such as *Rhodopseudomonas* was occasionally confirmed through more specific probes (Rhodopseud probe).

Fermented wastewater-fed PMCs showed a strong dominance of Alphaproteobacteria, followed by a balanced presence of classes such as Betaproteobacteria, Gammaproteobacteria and Deltaproteobacteria. Common to all cultures fed with real domestic wastewater was the high abundance of the PNSB *Rhodobacter* (GRb probe). In particular, cultures R1 to R3, operated under the PF regime, also had high numbers of *Rhodopseudomonas* and members of the Archaea domain (ARC915 probe). Desulfobulbaceae (sulphate reducers) and the aerobic bacteria *Amaricoccus* were also positively detected. These taxonomic groups found in R1 to R3 were mostly not detected in cultures R4 to R6, which were operated under a FF regime. In R4 to R6 an abundant presence of *Paracoccus* (PAR651) was found, while in R4 to R5 an abundant presence of *Zoogloea* (ZRA23a) and presence of *Thauera* (Thau832) were discovered. Despite *Zoogloea* and *Thauera* being members of the Rhodocyclaceae family, the probe RHC439 targeting this family did not indicate an abundant signal.

An important remark must be made on the high abundance of Gammaproteobacteria detected by FISH for both synthetic and real waste-fed cultures, which does not correlate with the values detected through amplicon sequencing. It is possible that sequencing may have underestimated the abundance of Gammaproteobacteria (similarly to what has been reported in other studies [39,40]), or that the higher biovolume of Gammaproteobacteria members (large rods and cocci) could have led to an overestimation of bacteria abundance through FISH.

### 3.3. Microscopic Observation: Cells Morphology through FISH and Nile Blue Staining

The microscopic observation of Nile blue-stained cultures allowed us to identify the morphology of the microorganisms that were capable of accumulating PHA. Likewise, from FISH observation of the same samples, it was possible to determine the morphology of some taxonomic groups. Coupling both forms of morphological information allowed, for some cultures to correlate PHA accumulation capacity to some taxonomic groups.

**Figure 1:**
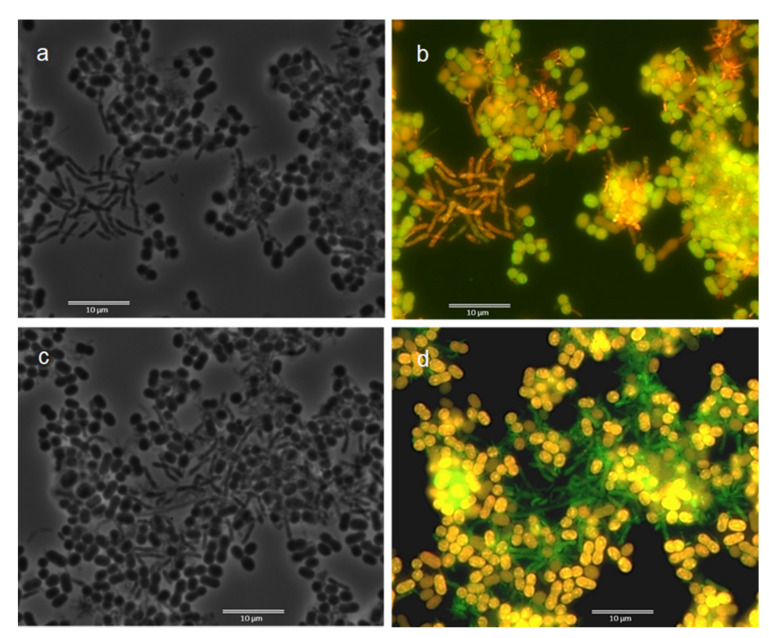
Microscopic images of culture S5 (synthetic medium, PF selection regime). (**a**,**c**)—Phase-contrast. (**b**)—Overlay image of bacteria hybridization with EUBmix and ALF969 (Alphaproteobacteria). (**d**)—Overlay image of bacteria hybridization with EUBmix and GAM42a (Gammaproteobacteria). Results for EUBmix are in green (FITC label) and, for specific probes, orange (Cy3 label).

For the synthetically fed cultures, two main morphotypes were detected by FISH observation, where most rods were identified as Alphaproteobacteria and cocci as Gammaproteobacteria, as exemplified in Figure 1 for culture S5.

**Figure 2:**
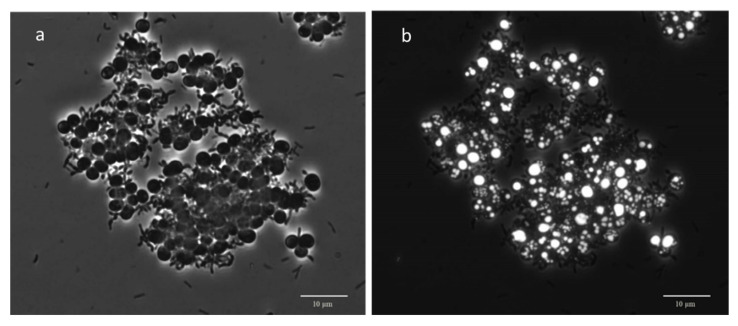
Microscopic images of the synthetically fed culture S5 operated under a permanent feast (PF) regime. (**a**)—Phase contrast; (**b**)—fluorescence image of Nile blue staining indicating PHA granules, mostly in cocci bacteria.

Nile blue staining indicates that both the rod and the cocci organisms were able to accumulate PHA in the FF-operated cultures S1 to S4 (exemplified in supplementary Figure S2 for cultures S3 and S4), while in culture S5 (PF regime), cocci were the main PHA accumulators (Figure 2). This strongly suggests that in S5, Gammaproteobacteria was the major bacterial group responsible for PHA accumulation.

In respect to the real waste-fed cultures (R1 to R6), a higher morphological diversity was observed amongst PHA producers and taxonomic groups (summarized in Table 5). Despite such diversity, for some cultures it was possible to attribute the PHA accumulation capacity to organisms of the Alphaproteobacteria (culture R6), Betaproteobacteria (culture R5), Gammaproteobacteria (culture R1, R2, R3) and Deltaproteobacteria classes (culture R2), based on Nile blue staining as shown in Supplementary Figure S3 and further discussed in Section 4.2.

## 4. Discussion

### 4.1. Synthetic Acetate Feeding (Cultures S1 to S5)

Cultures S1 to S5 were operated in closed reactors with Argon sparging and fed with synthetic acetate medium. This controlled feeding strategy allowed to evaluate the impact of operating conditions on the composition of the microbial community and link it with the PHA production performance of the systems. For cultures S1 and S2 (FF selection regimes), the cycle length was decreased from 24 h to 8 h (Table 1), which lowered the acetate concentration at the beginning of the cycle and decreased the Feast period length per cycle (considering that the OLR was kept the same). The resulting microbial changes were mostly observed within the Proteobacteria phylum. Although a similar content of Alphaproteobacteria was maintained in both cultures (S1—79%/(+++); S2—81%/(++); cf. Table S1 and Table 4), in S1, operated with 24 h cycles, the culture was equally enriched in microorganisms of the Bradyrhizobiaceae family and the *Blastochloris* genus (Table 2, S1—37.5% and 30.9%, respectively). In culture S2, with 8 h cycles, *Blastochloris* microorganisms prevailed (S2—60%). Furthermore, an increase in Xanthomonadaceae family members (to 10%) and a decrease in Chromatiaceae also occurred (Table 2). These results suggest that operation with lower acetate concentrations and/or shorter contact times with the substrate (shorter Feast phase), may favour the growth of Xanthomonadaceae and especially, *Blastochloris* bacteria, over both Bradyrhizobiaceae and Chromatiaceae family members. An increase in *Blastochloris* has also been reported for an acetate-fed PMC (designed for nutrient removal from wastewater) that was operated under a substrate competing environment [22]. Overall, these operating conditions and subsequent microbial community adaptation led to an S2 culture with faster metabolic kinetics, reflected in a polymer production rate 10 times higher (qPHA) (Table 1).

The existence of transient illumination periods is another factor that influenced the microbial community composition. Indeed, the culture in S3, which was operated under light/dark conditions as opposed to S2, which was permanently illuminated, enriched for members of the Rhizobiaceae family, which became dominant with an abundance over 70%, while all other taxonomic groups decreased in abundance (Table 2). In particular, *Blastochloris* abundance decreased from 60% in S2 to 13% in S3. Despite such a community composition change, the culture maintained its PHA production capacity, even improving its maximum PHA storage capacity in comparison to S2 (Table 1). This suggests that Rhizobiaceae members and/or *Blastochloris* comprised the organisms responsible for the PHA production.

Unlike the illumination conditions, an increase in the OLR that occurred from S2 to S4 (FF operated systems) did not seem to deeply affect the composition of the microbial community (Table 2), nor its polymer storage capacity. The culture maintained a similar kinetic behaviour regarding PHA production, with a higher PHA concentration in the system likely being due to the higher VSS resulting from the higher availability of carbon. Although some studies with aerobic PHA-accumulating MMC report a detrimental impact of increasing the OLR on the cultures’ PHA production performance [41], this was not observed in S4. This could be because the Feast-to-Famine length ratio still remained low (<0.13) [17], keeping the culture under selection pressure and, therefore, not altering the community.

A major change in the microbial culture was observed when a permanent carbon feast regime was implemented and the SRT was lowered from 6 to 3 days. Samples S1 and S5 were operated under different selection strategies with respect to carbon availability: S1 was under a feast and famine regime (FF), with alternated periods of presence and absence of carbon, and S5 was under the permanent feast regime (PF) with the constant presence of carbon. It seems that the higher/permanent presence of organic substrate combined with lower SRT did not favour Cyanobacteria (S1—5.1%; S5—0.36%; Table 2). Nor did those conditions favour microorganisms within the *Blastochloris* genus (S1—30.9%; S5—0.2%), which supports the previous suggestion that *Blastochloris* may be favoured under low acetate concentrations/short-time exposure to the substrate. Furthermore, under the acetate PF regime, Alphaproteobacteria abundance decreased (S1—79%; S5—59%; cf. Table S1) and became mostly represented by the Bradyrhizobiaceae family. In contrast, the abundance of Chromatiaceae increased (S1—2.2%; S5—26%). Therefore, from the combined results of sequencing, FISH and Nile blue staining, it can be suggested that in S5, under a PF regime and lower SRT, two main families were dominant, with rods belonging to the Bradyrhizobiaceae family and cocci belonging to the Chromatiaceae family. Chromatiaceae were the main bacterial group responsible for PHA production and presented a very high PHA storage capacity, considering that S5 attained a 60% gPHA/gVSS content and a PHA concentration of 75 Cmmol/L in the reactor (Table 1).

### 4.2. Fermented Real Waste Feeding (Cultures R1 to R6)

Cultures R1 to R6 were operated under 12 h light/12 h dark conditions and all were fed with a real waste feedstock consisting of a fermented mixture of domestic wastewater supplemented with 1% molasses. The feed was mostly composed of VFA (acetic, propionic, butyric and valeric acid) except for the R2 and R5 feedstock, which contained leftover sugars due to incomplete upstream feedstock fermentation [24].

#### 4.2.1. Permanent Feast Operation (R1 to R3)

Cultures R1 to R3 were subjected to a permanent feast regime, and despite the reactors being open, the constant carbon presence led to a very low microalgae content (thus minimal oxygen production) and resulted in anaerobic conditions [24]. Under this environment, community changes occurred due to variations in the OLR and fluctuations of the feedstock composition.

In R1, the culture was mostly comprised by Firmicutes, with *Trichococcus* dominating with an abundance over 68% (Table 3). This genus is often found in municipal sewage sludge and thrives in anaerobic environments through fermentative metabolism [42]. R1’s remaining populations were the PNSB *Rhodopseudomonas*, *Rhodobacter* and members of the PSB Chromatiaceae family, which combined, presented an overall abundance of ≈14% (Table 3) and may be responsible for the PHA accumulation in R1 (content of 20% gPHA/gVSS, cf. Table 1). It is likely that, within these purple bacteria, Chromatiaceae members were the major contributors to PHA production, since morphological observation of PHA producers through Nile blue staining indicated Gammaproteobacteria as the main responsible PHA storer (Table 5).

The impact of feedstock changes on the microbial community can be seen from R1 and R2. These cultures were operated under the same OLR, but R2’s feedstock contained sugar (20% of the total carbon) due to incomplete upstream fermentation of the feedstock (Table 1). The dominance of Firmicutes in R1 was replaced by Actinomycetales in R2 (>55% abundance, Table 3). Some microorganisms of Actinomycetales are often associated with gastrointestinal tracts [43] (which links to the domestic wastewater used as feedstock), and these may have found favourable fermentative conditions in the presence of sugar. The aforementioned purple bacteria of R1 were still present in R2, but in lower abundance, which correlates well with the low PHA content reported for R2 (4–6%, Table 1). These results show that, in PMC systems operated under an anaerobic environment, it is fundamental to ensure complete fermentation of the feedstock, thus preventing the dominance of fermentative organisms over purple bacteria and avoiding loss of PHA production capacity.

The decrease in the OLR is another factor that impacted the microbial culture composition in the systems fed with fermented wastewater. R3 was operated under low OLR with the permanent presence of carbon, but at low concentrations (<2 Cmmol/L) [24]. Considering the sequencing results, the culture became highly enriched in *Rhodopseudomonas* (>84% abundance), with a moderate presence of *Rhizobium* (11.2%), a minor presence of *Rhodobacter* (1.3%) and no Chromatiaceae detection (Table 3). However, the sequencing results do not agree with the FISH observations, which indicated an extreme abundance of *Rhodobacter* and the presence of *Amaricoccus*. While a sequencing underestimation of *Rhodobacter* may be a possibility (as mentioned in the results Section 3.2 for Gammaproteobacteria), the presence of *Amaricoccus* is less likely, since members of this genus are known for their strict aerobic metabolism [44] and oxygen was not available in the anaerobic system of R3. Despite these contrasting results, it is possible to say that Chromatiaceae members were outcompeted under conditions of low OLR and low carbon concentration. This agrees well with the previous results of S5, where Chromatiaceae became abundant under high-OLR conditions. Therefore, the PHA production capacity of R3 (up to 18% gPHA/gVSS, Table 1) was likely due to organisms within the genera *Rhodopseudomonas*, *Rhizobium* and/or *Rhodobacter*, which managed to prevail and store PHA under the stricter condition of low OLR/low carbon concentration.

#### 4.2.2. Feast and Famine Operation (R4 to R6)

Cultures R4 to R6 were operated under a feast and famine regime in an open system that, unlike cultures S1 to S5, was not sparged with an inert gas. During the feast phase, low oxidation reduction potential (ORP) levels were achieved in R4 to R6, while during the famine phase, the ORP would increase due to surface oxygen transfer and oxygen production by microalgae [24]. In cultures R4 to R6, the OLR gradually increased (Table 1), which extended the feast phase length, leading to fewer hours of oxygen availability during the famine phase. Sequencing results indicate that Firmicutes and Actinobacteria were mostly absent from these cultures—likely due to more oxidative conditions—with the exception of R5 (feedstock with 20% of the organic carbon as sugar), which contained an 11% abundance of the Actinobacteria *Propionicimonas* (Table 3). Members of this genus are facultative anaerobes that present better growth under anaerobic conditions and are able to ferment glucose into acetate and propionate [45]; thus, their growth was likely favoured by the presence of sugar in the feedstock. With the OLR increase, a population shift occurred within the Proteobacteria phylum. In R4 (low OLR, higher oxygen availability), *Paracoccus* bacteria were present in high abundance (53.7%/++), followed by *Rhodobacter* (15.4%/+++) and by diverse organisms from Alphaproteobacteria, Betaproteobacteria and Gammaproteobacteria, which presented low abundance (<6%). FISH results allowed the identification of some genera within the Betaproteobacteria class, namely *Thauera* (+) and *Zoogloea* (++). *Paracoccus*, *Thauera* and *Zoogloea* are aerobic bacteria that could have adapted to the higher oxidative conditions of R4, and additionally contribute to PHA production in R4 (6% gPHA/gVSS), since they have been extensively reported as PHA producers in aerobic MMC systems operated under FF regimes [18,19,46]. Despite their presence, higher PHA production values were not achieved in this system, possibly due to the low OLR.

In cultures R4 to R5, the OLR was increased, but the feedstock contained sugar and the community adapted with a strong decrease in *Paracoccus* and *Rhodobacter* abundance, down to 9.5% and 0.5%, respectively. On the other hand, the culture became highly enriched in Rhodocyclaceae (≈63%), and although FISH results indicated, again, the presence of the Rhodocyclaceae *Thauera* (+) and *Zoogloea* (++), the role of the Rhodocyclaceae, which became the dominant organisms, is unclear. Nevertheless, this new microbial community of R5 improved the PHA production performance in comparison to culture R4, which had a sugar-free feedstock (Table 1). This indicates that the higher oxygen availability in the FF-operated systems can prevent the dominance of fermentative organisms (unlike R2, with PF operation), allowing the presence of PHA producers and contributing to the system’s robustness in the face of fluctuating sugar levels in the feedstock.

In R6, the culture was operated with even higher OLR in comparison to R4, and the aerobic population was deeply affected. FISH showed that *Thauera* and *Zoogloea* became mostly non-existent, and the sequencing results showed that the abundance of *Paracoccus* fell to less than 1%. This is consistent with the new operating conditions leading to a more anaerobic environment that is adverse to aerobic organisms. *Rhodobacter* abundance also decreased, while the abundance of *Rhodopseudomonas*, *Rhizobium* and Hyphomicrobiaceae increased, suggesting their preference for more anaerobic conditions and/or higher OLR in FF regimes. No impact was seen on the abundance of Chromatiaceae members, which together with the aforementioned organisms, enabled a high production of PHA with a 31% gPHA/gVSS content (32 Cmmol PHA/L; 85 HB:15 HV) and a productivity of 2.67 gPHA/L.d, in an 8 h accumulation test (Table 1).

Finally, regarding the detection of Deltaproteobacteria organisms in cultures R4 to R6, DNA sequencing did not detect this class in any of the cultures, although FISH results indicated a high abundance of these organisms (Table 4). Furthermore, microscopic observations of R4 and R5 cultures identified a very specific morphology of swarm aggregated bacteria that were identified through FISH as Deltaproteobacteria and that presented a positive fluorescence signal for internal granules during Nile blue staining observations (Figure S3). More specific FISH probes indicated that these bacteria were not Desulfobacteraceae, Desulfobulbaceae or Desulfovibrionales (cf. Table 4). Based on their morphology, it can be hypothesized that the swarm aggregated bacteria are Myxococcales, an order of the Deltaproteobacteria that, under starvation conditions (such as the ones found in R4 and R5 FF systems), start to aggregate in swarming colonies and later differentiate into fruiting bodies for spore formation (Figure S4) [47]. Despite no PHA capability having been reported thus far (to the best of our knowledge) within this order, some members are able to produce lipid bodies during the developing phase, which cover a large part of the bacteria [48,49]. This probably explains the positive results for the Nile blue staining fluorescence observation (Table 5), which could be incorrectly attributed to the presence of PHA granules. Future work should employ specific probes for organisms within the Myxococcalales, such as CystbSUB1 and CystbSUB2 probes [37], which present specificity for Cystobacterineae (sub-order of Myxococcales), in order to verify the proposed hypothesis.

## 5. Conclusions

This work provided an overview of the bacterial communities that can be enriched in PMC systems designed for PHA production and operated either with synthetic acetate feed or real fermented feedstock, under different operating conditions. In addition, by overlapping the results from DNA sequencing, FISH, Nile blue staining and morphological observations, it was possible to find correlations between the system’s operating conditions, the microbial community and PHA performance. 

Within each selection strategy studied, PF and FF, the different operating conditions applied led to changes in the microbial community and culture performance. Overall, the results strongly suggest that Chromatiaceae members were the main PHA accumulators, with *Rhodopseudomonas*, *Rhodobacter* and *Rhizobium* also being capable of PHA production. It was also found that, when sugar is present in the feedstock, PF operated systems lose their capacity to produce PHA due to fermentative organisms (Actinomycetales) overcoming purple bacteria. On the contrary, FF systems are more robust to the presence of sugar, since the higher oxidative conditions prevent the dominance of fermentative organisms and maintain the presence of PHA producers. Therefore, if upstream feedstock fermentation can be strictly controlled, PF operation could be a very feasible option. PF operation can ensure conditions of high carbon concentration, which were shown to favour the growth of Chromatiaceae members, and thus, lead to greater PHA storage. If a FF operation system is implemented (e.g., due to uncomplete feedstock fermentation), an extended feast phase or higher OLR must be applied to obtain a PMC more enriched in phototrophic purple bacteria. These conditions favour the growth of *Rhodopseudomonas*, *Rhizobium* and Hyphomicrobiaceae, and lead to the presence of *Rhodobacter* and Chromatiaceae, creating a community capable of one of the highest PHA productivities reported thus far with real waste-fed PMC. Additionally, both PF and FF selection methods enabled the selection of PMCs with PHA production capacity under dark/light cycle operation, indicating the potential for future outdoor application under sunlight illumination. The results presented in this work show that phototrophic mixed cultures can evolve and adapt to different operating conditions, with a diversity of microorganisms of variable abundance being capable of contributing to PHA production. The identification of the microbial groups with higher PHA accumulation capacity, and knowledge of the operating conditions that maximize their presence and PHA storage, will help to define future operational strategies for further improvement of PHA production in phototrophic mixed microbial systems.

## Figures and Tables

**Table 1:** Summary of the operating conditions of the PHA-producing PMCs investigated in this study and corresponding biomass and PHA production performance. S1 to S5 are cultures fed with a synthetic medium and R1 to R6 are cultures fed with real fermented waste.

ID	Reactor	Feed	Selection	Operating Conditions				Culture Performance					Ref.
				Light	Cycle	SRT	OLR	VSS	SBR		Accumulation Test		
				(W/L)		(Days)	(Cmmol/L·d)	(g/L)	PHA Content ^a^	qPHA ^b^	PHA Content ^a^	qPHA ^b^	
S1	Closed, argon sparging, 30 °C	Synthetic acetate	FF	1.3	24 h light	6	10	1.1 ^c^	2–4 (4–8; 100 HB)	0.13 ± 0.03	n.d.	n.d.	-
S2					8 h light	6	10	1.4	0.5–2 (0.5–4; 100 HB)	1.2 ± 0.2	≈18 ^d^ (20; 100 HB)	1.1 ± 0.1	[15]
S3					4 h light + 4 h dark	6	10	0.7 ^c^	4–7 (12–17; 100 HB)	1.6 ± 0.1	15 ^e^ (30; 100 HB)	2.2 ± 0.1	[23]
S4					8 h light	6	20	2.0 ^c^	8–14 (8–12; 100 HB)	1.5 ± 0.1	22 ^f^ (18; 76 HB:24 HV)	n.d.	[17]
S5			PF	1.8	24 h light	3	40	1.2 ^c^	≈2 (4 -8; 100 HB)	0.05 ± 0.04	75 ^g^ (60; 100 HB)	0.73 ± 0.13	[16]
R0	Inoculum from high-rate algae ponds used for domestic wastewater treatment												[24]
R1	Open, 25 °C	Fermented mixture molasses + domestic wastewater	PF	1.1	12 h light + 12 h dark	6	12	1.7 ± 0.2	14 (20; 86 HB:14 HV)	0.13	n.d.	n.d.	
* R2						6	12.5	0.2	0.5–2 (4–6; ≈65 HB:35 HV)	n.d.	n.d.	n.d.	
R3						6	2–5	0.6 ± 0.2	7 (18; 60 HB:40 HV)	0.33	n.d.	n.d.	
R4			FF	1.9	12 h light + 12 h dark	6	2–5	0.9 ± 0.2	0.1–1.5 (0.5–6; ≈80 HB:20 HV)	0.42	n.d.	n.d.	
* R5						6	7	1.0 ± 0.2	0.3–6 (0.5–13; ≈40 HB:60 HV)	n.d.	n.d.	n.d.	
R6						6	7–10	1.6 ± 0.2	1–6 (4–9; 63 HB:37 HV)	0.62	32 ^h^ (31; 85 HB:15 HV)	1.84	

FF—Feast and Famine; PF—Permanent Feast; HB—hydroxybutyrate monomer; HV—hydroxyvalerate monomer; * high sugar content in the feedstock (sugar accounted for 20% of the total carbon); ^a^ PHA content in Cmmol/L (% g PHA/g VSS; HB:HV ratio on a C-mol basis); ^b^ qPHA in Cmmol/Cmmol X·d; ^c^ active biomass (g X/L); ^d^ accumulation batch test with 4 pulses addition of acetate (13 h test); ^e^ accumulation batch test with 6 pulses addition of acetate (8 h test); ^f^ batch test with 4 pulses mixture addition of acetate, butyrate, propionate (8 h test); ^g^ SBR test at higher light intensity (72 h—6.2 W/L); ^h^ accumulation batch test with multiple pulses addition and higher light intensity (8 h—6.2 W/L); n.d.—not determined.

**Table 2:** Heatmap of the most abundant taxonomic groups (≥2% of abundance in at least one sample) identified in the PMCs fed with acetate synthetic medium 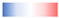 less abundant to more abundant.

					S1	S2	S3	S4	S5
Phylum	Class	Order	Family	Genus	%				
Actinobacteria	-	-	-	-	0.0	2.1	0.0	0.1	1.8
Bacteroidetes	-	-	-	-	5.8	0.1	0.2	0.1	7.4
Chloroflexi	-	-	-	-	2.2	0.1	0.4	0.1	0.0
Cyanobacteria	-	-	-	-	5.1	4.6	6.6	1.7	0.4
Proteobacteria	Alpha proteobacteria	Rhizobiales	Bradyrhizobiaceae	-	37.5	6.5	1.6	5.1	58.6
			Phyllobacteriaceae	-	0.0	1.0	0.0	2.0	0.0
			Rhizobiaceae	-	0.1	0.5	72.5	0.0	0.1
			Hyphomicrobiaceae	*Blastochloris*	30.9	60.0	12.7	74.7	0.2
		Rhodobacterales	Rhodobacteraceae	-	7.7	5.2	0.2	0.2	0.0
		Rickettsiales	-	-	0.0	2.7	0.0	0.3	0.0
	Gamma proteobacteria	Chromatiales	Chromatiaceae	-	2.2	0.0	0.3	5.1	26.0
		Xanthomonadales	Xanthomonadaceae	-	0.0	10.1	0.5	0.6	0.0
Other phyla	-	-	-	-	3.5	0.1	0.6	0.8	0.1
Total					95.0	93.1	95.6	90.9	94.6

**Table 3:** Heatmap of the most abundant taxonomic groups (≥2% of abundance in at least one sample) identified in PMCs fed with a fermented mixture of molasses and domestic wastewater. 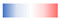 less abundant to more abundant.

					R0	R1	R2	R3	R4	R5	R6
Phylum	Class	Order	Family	Genus	%						
Actinobacteria	Actinobacteridae	Actinomycetales	-	-	0.5	0.0	55.2	0.1	0.0	0.2	0.1
	unknown	Corynebacteriales	-	-	4.3	0.0	0.0	0.0	0.2	0.0	0.0
		Propionibacteriales	Propionibacteriaceae	*Propionicimonas*	0.0	0.0	0.8	0.2	0.1	11.1	0.4
		Micrococcales	-	-	2.1	0.0	0.1	0.0	0.6	0.3	0.9
		PeM15	-	-	10.9	0.0	0.0	0.0	0.1	0.1	0.0
Chloroflexi	Caldilineae	Caldilineales	-	-	11.7	0.0	0.0	0.0	0.8	0.3	0.0
Cyanobacteria	-	-	-	-	0.3	0.0	0.4	1.0	0.4	1.4	2.4
Firmicutes	Bacilli	Lactobacillales	Carnobacteriaceae	*Trichococcus*	0.6	68.8	3.5	0.0	0.0	0.0	0.0
	Clostridia	Clostridiales	Clostridiaceae	*Clostridium*	2.0	0.3	1.2	0.0	0.3	0.1	0.0
			Peptostreptococcaceae	-	6.7	10.0	2.0	0.0	0.3	0.1	0.0
Proteobacteria	Alpha proteobacteria	Rhizobiales	Bradyrhizobiaceae	*Rhodopseudomonas*	7.2	2.7	8.7	84.4	1.2	1.2	10.9
			Rhizobiaceae	*Rhizobium*	0.6	0.0	0.0	11.2	1.0	1.9	32.9
			Hyphomicrobiaceae	-	3.0	0.1	0.8	0.1	1.8	0.5	22.5
		Rhodobacterales	Rhodobacteraceae	*Rhodobacter*	5.7	6.7	1.5	1.3	15.4	0.5	5.7
				*Paracoccus*	0.5	0.0	0.1	0.0	53.7	9.5	0.7
		Caulobacterales	-	-	0.8	0.4	0.0	0.0	1.0	3.7	0.0
	Beta proteobacteria	Burkholderiales	Comamonadaceae	-	3.6	0.0	0.0	0.3	0.5	0.2	0.1
		Rhodocyclales	Rhodocyclaceae	-	1.6	0.0	0.1	0.1	4.4	62.9	0.1
	Gamma proteobacteria	Chromatiales	Chromatiaceae	-	0.4	5.0	2.1	0.0	2.9	0.1	3.9
		Xanthomonadales	-	-	1.7	0.1	0.0	0.0	5.9	0.3	0.5
Other phyla	-	-	-	-	4.1	0.2	2.0	0.1	1.8	1.0	1.8
Total					68.3	94.3	78.5	98.8	92.4	95.4	82.9

**Table 4:** FISH results for the PMCs fed with acetate synthetic medium (S1 to S5) and PMC fed with a fermented mixture of domestic wastewater supplemented with molasses (R1 to R6).

Samples	ALF969	BET42a	GAM42a	Delta495a	ARC915	LGC0354	GRb	RHC439	Rhodo-2	Rhodopseud	DSBAC357	DSB706	DSV687	PAR651	Azo644	Thau832	ZRA23a	AMAR839	Ref.
S1	+++	±	++	n.a.	n.a.	n.a.	n.a.	n.a.	n.a.	++	n.a	n.a	n.a	n.a	n.a	n.a	n.a	n.a	*
S2	++	-	+++	-	-	n.a	+	-	+	+	n.a.	n.a.	n.a.	-	-	-	-	-	[15]
S3	++	-	++	-	n.a	n.a	n.a	n.a	n.a	-	n.a	n.a	n.a	n.a	n.a	n.a	n.a	n.a	[23]
S4	++	-	+++	n.a	n.a	n.a	-	n.a	-	+	n.a	n.a	n.a	n.a	n.a	n.a	n.a	n.a	[17]
S5	++	-	++	n.a	n.a	n.a	n.a	n.a	n.a	-	n.a	n.a	n.a	n.a	n.a	n.a	n.a	n.a	[16]
R1	inc.	±	++	±	++	±	+	-	-	++	-	±	-	±	+	+	±	±	*
R2	inc.	-	++	+	+	±	inc.	-	-	+	±	±	-	±	-	-	±	+	
R3	+++	++	+	++	++	±	+++	±	-	++	-	+	-	±	-	-	-	+	
R4	+++	++	++	++	±	±	+++	±	-	±	±	+	±	++	-	+	++	±	
R5	++	++	+	+++	±	±	++	±	-	±	±	±	±	++	±	+	++	-	
R6	++	+	+	++	-	-	++	±	-	-	-	+	±	++	-	±	-	±	

Extremely abundant (+++); abundant (++); present (+); almost non-existent (±); not detected (-); n.a.—not analysed; inc.—inconclusive. * This study.

**Table 5:** Results from the microscopic observation of the real waste-fed cultures (R1 to R6) indicating the morphology of organisms with positive fluorescence signal under Nile blue staining (indication of possible PHA accumulators) and the most dominant morphology of bacteria from the Alphaproteobacteria, Betaproteobacteria, Gammaproteobacteria and Deltaproteobacteria classes (observed through FISH).

	Morphology from Nile Blue Observation	Morphology from FISH Observation					
		ALF969		BET42a		GAM42a	Delta495a
R1	Coccus (large)	^a^	Rods (short)	Rods (thin)		Coccus	Not present
R2	Coccus (aggregated in rectangular sets) Rods (thin); Rods (thick)	^a^	Rods (short)	Not present		Rods (thick) Coccus	Rods (thin)
R3	Rods (long and thick)	Rods (short and thick) Rods (thin)		Rods (short and thick)		Rods (long and thick)	Rods (thin) Ovoid
R4	Rods (aggregated in swarms) *	Rods Ovoid		Rods (short and thin)		Rods (long and thick)	Rods (swarms)
R5	Coccus Rods (aggregated in swarms) *	Rods (short and thick)		^b^	Coccus (large)	Rods (short)	Rods (swarms)
R6	Coccus (large) Rods (short)	Coccus		Rods		Rods	Rods

* Possibly lipid bodies, see discussion in Section 4.2; ^a^ morphology from observation with GRb probe; ^b^ morphology from observation with Thau832 probe.

## Data Availability

The data presented in this study are contained within the article and supplementary materials, being available upon request from the corresponding author.

## Supplementary Materials

The following are available online at https://www.mdpi.com/article/10.3390/microorganisms10020351/s1, Figure S1: FISH results for the PMC fed with a fermented mixture of domestic wastewater supplemented with molasses (R1 to R6) presented in a radar chart, Figure S2: Microscopic images of the synthetically fed cultures S3 and S4 operated under a feast and famine (FF) regime, Figure S3: Fluorescence images of Nile blue staining indicating polyhydroxyalkanoates granules in R1 to R6 cultures, Figure S4: Microscopic pictures of swarm shaped aggregation observed in culture R5, Table S1: DNA sequencing results of the phototrophic mixed cultures operated with synthetic acetate, Table S2: DNA sequencing results of the phototrophic mixed cultures operated with fermented wastewaters with molasses, Table S3: FISH results for GAOmix and PAOmix probes for the PMC fed with acetate synthetic medium (S1 to S5) and PMC fed with a fermented wastewater (R1 to R6).
