# Supplementary material for: Dynamics of Microbial Communities in Phototrophic Polyhydroxyalkanoate Accumulating Cultures
Source: Microorganisms. 2022 Feb 3;10(2):351. doi: 10.3390/microorganisms10020351 (PMC8874877; doi:10.3390/microorganisms10020351)
Supplement: Supplementary file 1 [file microorganisms-10-00351-s001.zip › microorganisms-1555926-supplementary.pdf]

Supplementary Material

Table S1. DNA sequencing results of the phototrophic mixed cultures operated with synthetic acetate.

	S1	S2	S3	S4	S5
	%				
p_Acidobacteria/c_Chloracidobacteria	0.00	0.04	0.00	0.00	0.00
p_Actinobacteria	0.00	2.10	0.00	0.06	1.80
o_Actinomycetales	0.00	2.10	0.00	0.06	1.80
p_Bacteroidetes	5.80	0.08	0.20	0.14	7.36
c_Bacteroidia/o_Bacteroidales	3.60	0.00	0.10	0.06	7.08
c_Shingobacteriia/o_Saprospirales/f_Saprospiraceae	0.00	0.06	0.00	0.00	0.00
c_Cytophagales/ f__WCHB1−69_OTU_43;17;	2.20	0.00	0.10	0.00	0.00
p_Chlorobi	0.10	0.00	0.60	0.76	0.00
p_Chloroflexi	2.20	0.06	0.40	0.10	0.00
c_Anaerolineae	2.20	0.04	0.10	0.10	0.00
c_Thermomicrobia	0.00	0.02	0.00	0.00	0.00
c__Ktedonobacteria_OTU_24	0.00	0.00	0.30	0.00	0.00
p_Cyanobacteria/c_Chloroplast	5.10	4.62	6.60	1.70	0.36
p_Firmicutes	0.00	0.04	0.00	0.00	0.12
c_Bacilli	0.00	0.04	0.00	0.00	0.00
c_Clostridia	0.00	0.00	0.00	0.00	0.12
p_Proteobacteria	80.60	91.22	88.00	90.52	85.58
c_Alphaproteobacteria	77.70	80.96	87.10	84.32	59.46
o_Rhizobiales	68.60	68.58	86.80	81.92	59.08
f_Bradyrhizobiaceae	37.50	6.54	1.60	5.08	58.64
f_Methylocystaceae	0.10	0.54	0.00	0.10	0.12
f_Phyllobacteriaceae	0.00	1.00	0.00	1.98	0.00
f_Rhizobiaceae	0.10	0.52	72.50	0.02	0.12
f_Hyphomicrobiaceae/g_Blastochloris	30.90	59.98	12.70	74.74	0.20
o_Rhodobacterales/f_Rhodobacteraceae	7.70	5.24	0.20	0.22	0.00
g_Pannonibacter	1.40	0.00	0.10	0.00	0.00
o_Rhodospirillales/f_Rhodopirillaceae/g_Telmatospirillum	0.00	0.00	0.00	0.00	0.06
o_Rickettsiales	0.00	2.74	0.00	0.26	0.00
o_Sphingomonadales/f_Sphingomonadaceae/g_Sphingopyxis	0.00	0.04	0.00	0.20	0.00
c_Betaproteobacteria	0.60	0.08	0.10	0.00	0.04
o_Burkholderiales/f_Comamonadaceae/g_Comamonas	0.00	0.06	0.00	0.00	0.00
o_Burkholderiales/f_Comamonadaceae/g_Acidovorax	0.60	0.00	0.10	0.00	0.00
o_Rhodocyclales/f_Rhodocyclaceae	0.00	0.02	0.00	0.10	0.04
c_Gammaproteobacteria	2.30	10.18	0.80	6.20	26.06
o_Chromatiales/f_Chromatiaceae	2.20	0.00	0.30	5.12	26.02
o_Xanthomonadales/f_Xanthomonadaceae	0.00	10.12	0.50	0.64	0.02
p_Verrucomicrobia/c_/o_/f_/g_Luteolibacter	0.00	0.00	0.00	0.04	0.00
p_Tenericutes/c_/o_/f_/g_Acholeplasma	3.40	0.00	0.00	0.00	0.00
Total	**97.20**	**98.16**	**95.80**	**93.32**	**95.22**

Table S2. DNA sequencing results of the phototrophic mixed cultures operated with fermented wastewaters with molasses.

	R0	R1	R2	R3	R4	R5	R6
	%						
p_Acidobacteria/c_Chloracidobacteria	0.0	0.0	0.1	0.0	0.0	0.0	0.1
p_Actinobacteria	19.7	0.2	56.5	0.3	1.3	12.1	2.6
o_Actinomycetales	0.5	0.0	55.2	0.1	0.0	0.2	0.2
f_Actinomycetaceae	0.5	0.0	55.2	0.1	0.0	0.2	0.1
g_Actinomyces	0.2	0.0	55.2	0.1	0.0	0.1	0.1
f_Corynebacteriaceae	0.1	0.0	0.0	0.0	0.0	0.0	0.0
f_Nocardiaceae	0.2	0.0	0.0	0.0	0.0	0.0	0.0
o_Coriobacteriales	0.1	0.1	0.1	0.0	0.0	0.0	0.8
o_Corynebacteriales	4.3	0.0	0.0	0.0	0.2	0.0	0.0
o_Mycobacteriales	0.2	0.0	0.0	0.0	0.0	0.0	0.0
o_Propionibacteriales	0.6	0.1	0.9	0.2	0.3	11.5	0.8
f_Propionibacteriaceae / g_Propionicimonas	0.0	0.0	0.8	0.2	0.1	11.1	0.4
o_Micrococcales	2.1	0.0	0.1	0.0	0.6	0.3	0.9
o_PeM15	10.9	0.0	0.0	0.0	0.1	0.1	0.0
p_Bacteroidetes	0.6	0.2	1.5	0.1	1.1	1.0	0.9
c_Bacteroidia/o_Bacteroidales	0.0	0.0	0.8	0.1	0.0	0.0	0.1
f_Prevotellaceae / g_Prevotella	0.0	0.0	0.0	0.0	0.0	0.0	0.1
c_Flavobacteriia	0.4	0.1	0.5	0.0	0.4	0.0	0.5
c_Cytophagales	0.0	0.0	0.0	0.0	0.4	0.5	0.0
p_Chlorobi / c_Chlorobia / o_Chlorobiales / f_Chlorobiaceae	0.0	0.0	0.0	0.0	0.0	0.0	1.3
p_Chloroflexi	13.7	0.3	1.1	0.0	1.0	0.6	0.1
c_Anaerolineae	0.3	0.2	0.1	0.0	0.1	0.2	0.0
c__Caldilineae / o_Caldilineales	11.7	0.0	0.0	0.0	0.8	0.3	0.0
p_Cyanobacteria/c_Chloroplast	0.3	0.0	0.4	1.0	0.4	1.4	2.4
p_Firmicutes	10.8	83.6	19.7	0.5	1.7	0.4	2.4
c_Bacilli	1.3	72.0	12.1	0.3	0.9	0.2	0.3
o_Lactobacillales	1.2	71.5	11.6	0.3	0.1	0.2	0.3
f_Carnobacteriaceae/g_Trichococcus	0.6	68.8	3.5	0.0	0.0	0.0	0.0
f_Lactobacillaceae/g_Leuconostoc	0.0	0.9	0.0	0.0	0.0	0.0	0.0
c_Clostridia	9.1	11.8	7.0	0.1	0.7	0.2	2.0
o_Clostridiales	9.1	11.8	7.0	0.1	0.7	0.2	2.0
f_Clostridiaceae / g_Clostridium	2.0	0.3	1.2	0.0	0.3	0.1	0.0
f_Veillonellaceae	0.0	0.0	0.6	0.0	0.0	0.0	0.1
f_Peptostreptococcaceae	6.7	10.0	2.0	0.0	0.3	0.1	0.0
f_Ruminococcaceae	0.0	0.0	0.4	0.0	0.0	0.0	0.3
c_Negativicutes / o_Selenomonadales / f__Acidaminococcaceae_OTU_21	0.0	0.0	0.6	0.0	0.0	0.0	0.1

Table S2. *Cont.*

	R0	R1	R2	R3	R4	R5	R6
	%						
p_Proteobacteria	50.8	15.4	19.6	98.0	93.5	84.3	88.4
c_Alphaproteobacteria	42.3	10.1	12.8	97.6	79.5	20.8	81.9
o_Rhizobiales	21.1	2.9	9.7	95.9	7.6	6.5	73.7
f_Bradyrhizobiaceae	7.2	2.7	8.7	84.4	1.2	1.2	10.9
g_Rhodopseudomonas	5.7	2.7	8.7	83.9	0.7	0.7	10.5
f_Rhizobiaceae	0.6	0.0	0.0	11.2	1.0	1.9	32.9
g_Rhizobium	0.5	0.0	0.0	11.2	0.6	1.7	32.8
f_Hyphomicrobiaceae	3.0	0.1	0.8	0.1	1.8	0.5	22.5
g_Blastochloris	0.1	0.0	0.3	0.0	0.0	0.0	0.3
o_Rhodobacterales	18.4	6.8	1.6	1.3	70.6	10.0	6.3
f_Rhodobacteraceae	18.4	6.8	1.6	1.3	70.6	10.0	6.3
g_Rhodobacter	6.5	6.7	1.5	1.3	15.4	0.5	5.7
g_Paracoccus	0.5	0.0	0.1	0.0	53.7	9.5	0.7
g_Amaricoccus	0.0	0.0	0.0	0.0	0.1	0.0	0.0
o_Rhodospirillales	0.5	0.1	1.4	0.3	0.3	0.6	1.8
f_Rhodospirillaceae	0.1	0.0	0.1	0.2	0.1	0.2	0.1
o_Caulobacterales	0.8	0.4	0.0	0.0	1.0	3.7	0.0
c_Betaproteobacteria	5.3	0.1	0.2	0.4	5.0	63.0	1.9
o_Burkholderiales	3.7	0.0	0.1	0.3	0.5	0.2	1.8
f_Comamonadaceae	3.6	0.0	0.0	0.3	0.5	0.2	0.1
o_Rhodocyclales/f_Rhodocyclaceae	1.6	0.0	0.1	0.1	4.4	62.9	0.1
g_OTU-10	0.0	0.0	0.0	0.0	4.3	58.1	0.0
c_Gammaproteobacteria	2.7	5.2	6.7	0.0	8.9	0.5	4.4
o_Chromatiales/f_Chromatiaceae	0.4	5.0	2.1	0.0	2.9	0.1	3.9
o_Xanthomonadales	1.7	0.1	0.0	0.0	5.9	0.3	0.5
p_Saccharibacteria	2.0	0.0	0.4	0.0	0.0	0.0	0.8
p_Planctomycetes / c_Planctomycetacia	1.4	0.0	0.0	0.0	0.7	0.0	0.0
p_Synergistetes	0.1	0.0	0.0	0.0	0.0	0.0	0.0
Total	**99.4**	**99.7**	**99.3**	**99.9**	**99.7**	**99.8**	**99.0**

Table S3. FISH results for GAOmix and PAOmix probes for the PMC fed with acetate synthetic medium (S1 to S5) and PMC fed with a fermented wastewater (R1 to R6).

Samples	GAOmix (GBG2 + GAOQ989)	PAOmix (PAO462 + PAO651 + PAO846)
**S1**	n.a	n.a
**S2**	(-)	(-)
**S3**	(-)	n.a
**S4**	n.a	n.a
**S5**	n.a	n.a
**R1**	(-)	(-)
**R2**	(-)	(-)
**R3**	(-)	(-)
**R4**	(-)	(-)
**R5**	(-)	(-)
**R6**	(-)	(-)

Non-present (-); n.a. – not analyzed

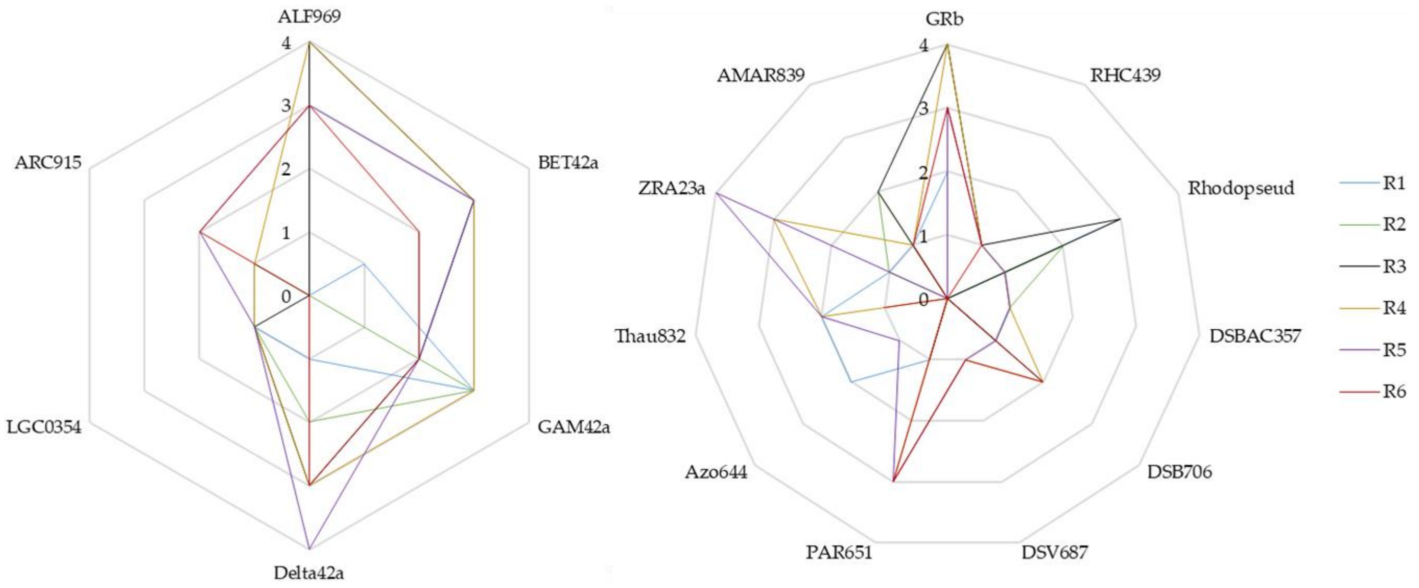

Figure S1: FISH results for the PMC fed with a fermented mixture of domestic wastewater supplemented with molasses (R1 to R6) presented in a radar chart. The relative abundances correspond to **4 -** Extremely abundant (+++), **3 -** Abundant (++), **2 -** Present (+), **1 -** Almost non-existent (±) and **0 -** Not detected, not analysed or inconclusive.

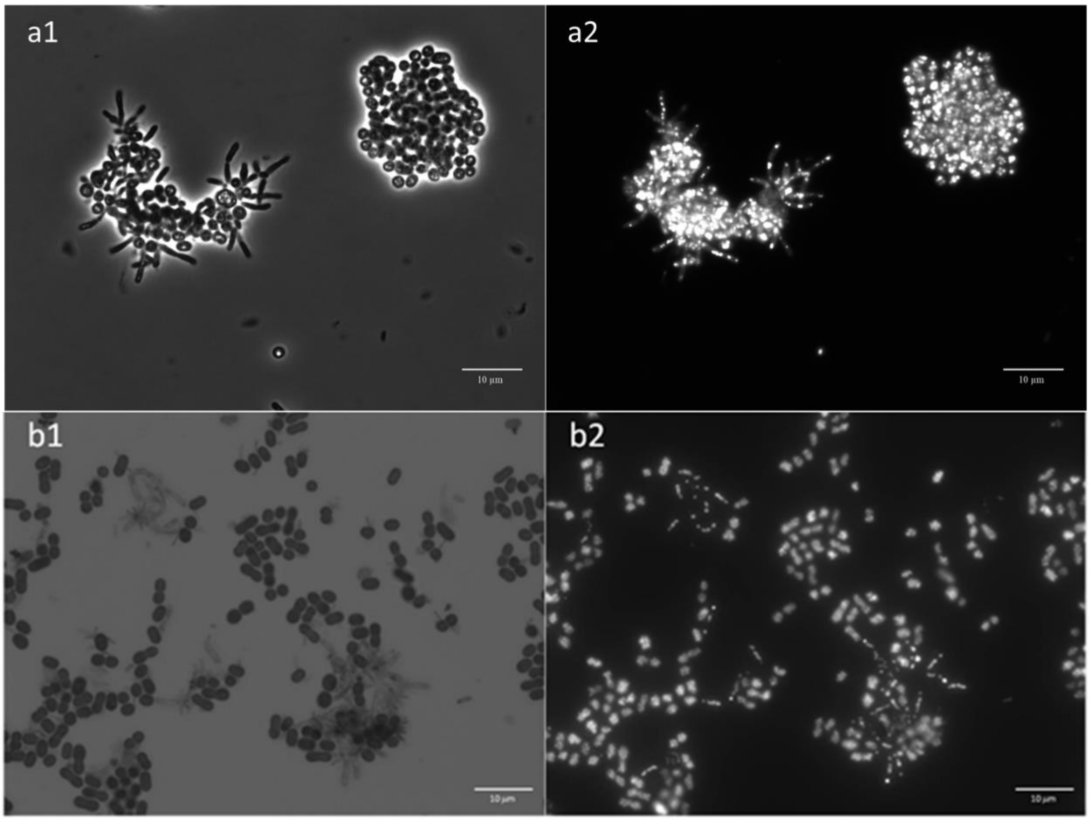

Figure S2. Microscopic images of the synthetically fed cultures S3 and S4 operated under a feast and famine (FF) regime. **a1** – Phase contrast of culture S4; **b1** – Bright field of culture S3; **a2 and b2** – Fluorescence images of Nile blue staining indicating PHA granules in in culture S4 and S3, respectively. In the two cultures, both rods and cocci bacteria present PHA granules.

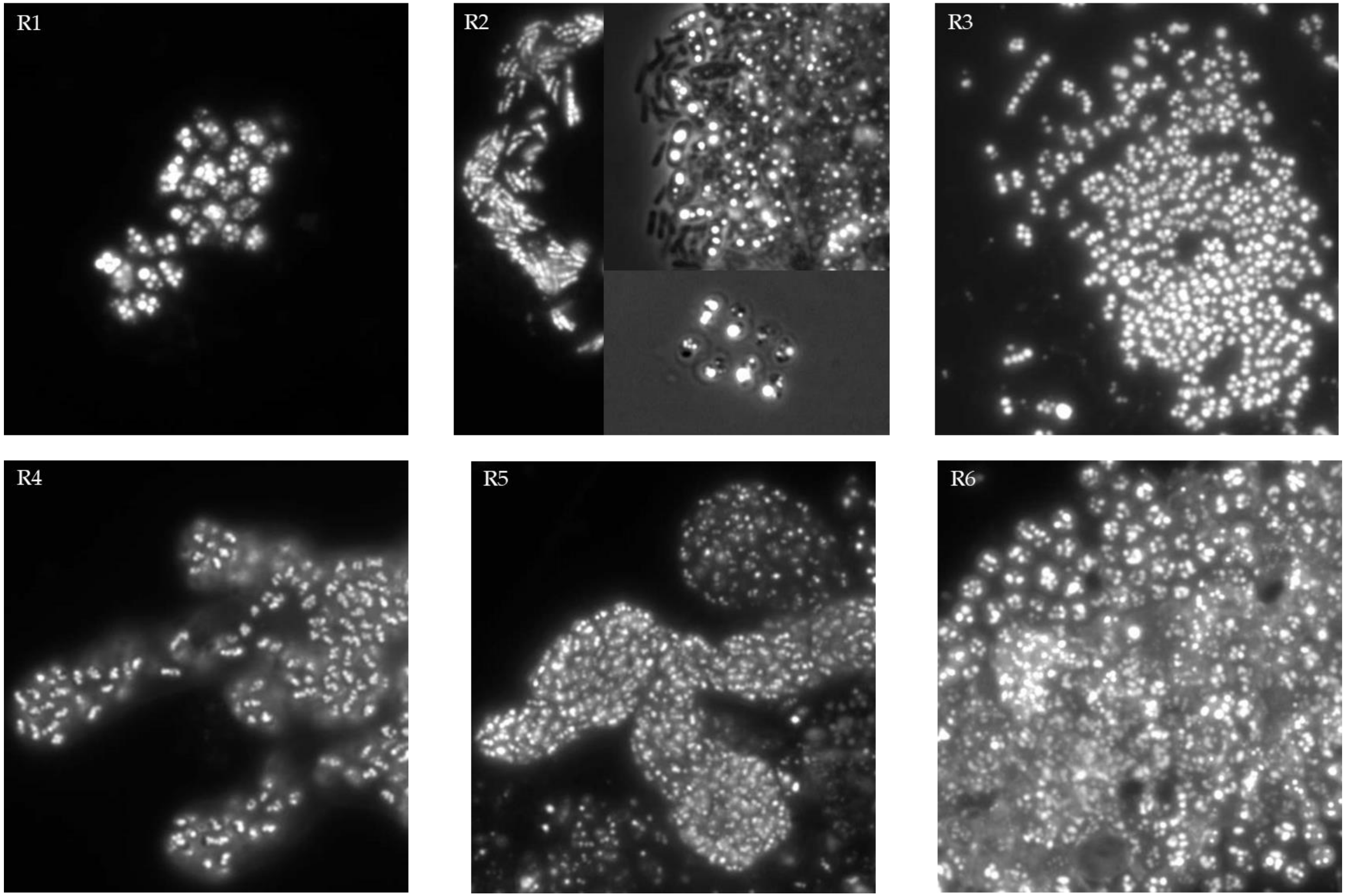

Figure S3. Fluorescence images of Nile blue staining indicating polyhydroxyalkanoates granules in R1 to R6 cultures. Magnification of 100x.

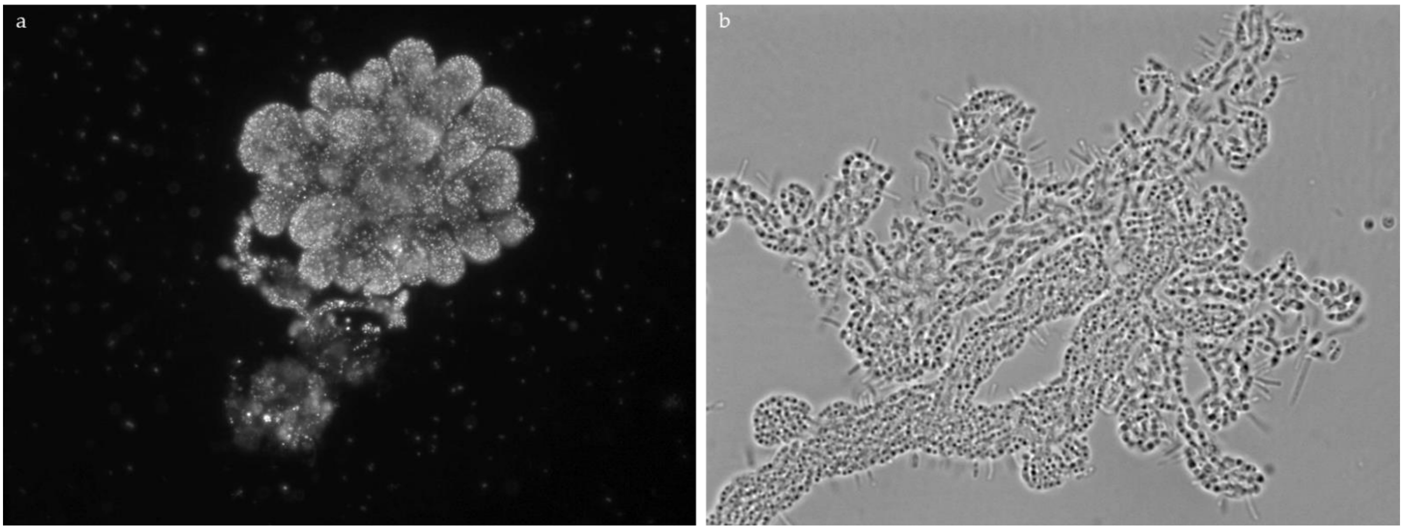

Figure S4. Microscopic pictures of swarm shaped aggregation observed in culture R5. **a –** Fruituig body development observation with Nile blue staining, magnification 100x; **b** – Bright field observation of swarm aggregated bacteria identified by FISH as Deltaproteobacteria, magnification 100x.
